# Metabolite Profiling Reveals the Effect of Cold Storage on Primary Metabolism in Nectarine Varieties with Contrasting Mealiness

**DOI:** 10.3390/plants12040766

**Published:** 2023-02-08

**Authors:** Patricio Olmedo, Baltasar Zepeda, Joaquín Delgado-Rioseco, Carol Leiva, Adrián A. Moreno, Karen Sagredo, Francisca Blanco-Herrera, Romina Pedreschi, Rodrigo Infante, Claudio Meneses, Reinaldo Campos-Vargas

**Affiliations:** 1 Centro de Estudios Postcosecha, Facultad de Ciencias Agronómicas, Universidad de Chile, Santiago 8831314, Chile; 2 Horticulture and Product Physiology, Department of Plant Sciences, Wageningen University, P.O. Box 16, 6700 AA Wageningen, The Netherlands; 3 Centro de Biotecnología Vegetal, Facultad de Ciencias de la Vida, Universidad Andrés Bello, Santiago 8370186, Chile; 4 Departamento de Producción Agrícola, Facultad de Ciencias Agronómicas, Universidad de Chile, Santiago 8831314, Chile; 5 ANID—Millennium Science Initiative Program—Millennium Nucleus for the Development of Super Adaptable Plants (MN-SAP), Santiago 8370186, Chile; 6 Escuela de Agronomía, Facultad de Ciencias Agronómicas y de los Alimentos, Pontificia Universidad Católica de Valparaíso, Quillota 2260000, Chile; 7 Millennium Institute Center for Genome Regulation (CRG), Santiago 7800003, Chile; 8 Departamento de Fruticultura y Enología, Facultad de Agronomía e Ingeniería Forestal, Pontificia Universidad Católica de Chile, Santiago 7820436, Chile; 9 Departamento de Genética Molecular y Microbiología, Facultad de Ciencias Biológicas, Pontificia Universidad Católica de Chile, Santiago 8331150, Chile

**Keywords:** chilling injury, postharvest, primary metabolism, *Prunus persica*, wooliness

## Abstract

Chilling injury is a physiological disorder caused by cold storage in peaches and nectarines. The main symptom of chilling injury is mealiness/wooliness, described as a lack of juice in fruit flesh. In this work, we studied two nectarine varieties (Andes Nec-2 and Andes Nec-3) with contrasting susceptibility to mealiness after cold storage. A non-targeted metabolomic analysis was conducted by GC-MS to understand if changes in metabolite abundance are associated with nectarine mealiness induced by cold storage. Multivariate analyses indicated that in unripe nectarines, cold storage promoted a higher accumulation of amino acids in both varieties. Interestingly, for ripe nectarines, cold storage induced an accumulation of fewer amino acids in both varieties and showed an increased abundance of sugars and organic acids. A pathway reconstruction of primary metabolism revealed that in ripe nectarines, cold storage disrupted metabolite abundance in sugar metabolism and the TCA cycle, leading to a differential accumulation of amino acids, organic acids, and sugars in mealy and juicy nectarines.

## 1. Introduction

Nectarines (*Prunus persica* (L.) var. nectarina) and peaches (*Prunus persica* (L.) Batsch) are some of the most important fruits of the Rosaceae family [1]. Currently, there is a wide diversity of nectarines and peaches, introducing more than 1000 new varieties during the last decades [2]. Nectarines are climacteric stone fruits and contain a lignified endocarp enclosing the seed, a fleshy mesocarp, and a thin epicarp. Additionally, the nectarine skin lacks trichomes [3]. These fruits are an important source of vitamin C (ascorbic acid), carotenoids and several phenolic compounds, giving them an elevated nutritional value in the fresh fruit market [4,5].

Once ripened, nectarines quickly deteriorate at ambient temperature and to avoid this senescent process, cold storage at non-freezing temperatures is required to slow down the decay development [6]. Nonetheless, refrigeration limits the postharvest fruit life by triggering several physiological disorders known as chilling injury [7]. Chilling injury is observed on ripened fruits after the removal from cold storage and is characterized by the reduction of quality parameters of the fruit with a consequent loss of consumer acceptance [6,8]. Common chilling injury symptoms are flesh bleeding, flesh browning, and mealiness/wooliness [6,9]. Mealiness is manifested as a lack of crispiness, decreased hardness and low juice content of the mesocarp, affecting the texture and organoleptic properties of the fruit [6,8,10,11]. 

Mealiness has been associated with cell wall disassembly, particularly in the pectic domain. In mealy fruits, altered homogalacturonan and rhamnogalacturonan I induce the formation of gel structures within intracellular spaces, leading to a reduction in cell–cell adhesion [12,13]. In addition, mealiness has been related to a reduction in the respiration rate and ethylene production, affecting many ripening processes [14]. Cell wall-associated enzyme activities, such as polygalacturonases, require an initiation step mediated by ethylene, driving to an abnormal flesh softening in mealy fruits [13,14]. Transcriptomic analyses also pointed out to an increased expression of cell wall-associated genes in juicy nectarines, such as cellulose synthase, cellulase, β-xylosidase, pectate lyase, and polygalacturonase [15,16]. Proteomic analyses have been described chilling injury differential protein accumulation in peach fruits associated with cell wall dynamics, including pectin methylesterase, polygalacturonase [17], and with carbon metabolism, such as α-galactosidase, galactose dehydrogenase and NADP-dependent malic enzyme [18]. 

During abiotic stresses, plant cells promote the influx and biosynthesis of different organic osmolytes, such as sugars, polyamines, secondary metabolites, methylamines, polyols, and amino acids, thus accumulating them for homeostasis maintenance [19,20]. Cold storage and low temperature stress can trigger processes of dehydration-associated responses in plant tissues [21]. As a protective response, plants can modify their metabolic profile, allowing the accumulation of osmoregulation substances to prevent cold damage [21]. These metabolic shifts are mainly derived from alterations in central carbon metabolism, involving glycolysis and the tricarboxylic acid cycle (TCA cycle) as a source of carbon skeletons for different metabolite biosynthesis [22].

Metabolomic analyses include the study of compounds involved in different plant cell metabolisms and support understanding many biological processes. However, there still needs to be more information on the Rosaceae family, particularly the genus *Prunus* [23]. Therefore, those works have been carried out using mainly peaches and have focused on describing the metabolic differences associated with harvest date [1], comparison of varieties at harvest [24], fruit growth and development [25], fruit ripening after cold storage [26,27,28,29], and treatments affecting quality traits after cold storage [30,31,32,33]. Thus, this work aimed to identify the changes in primary metabolism induced by the effect of cold storage on immature nectarines and the changes associated with the ripening process on shelf life, where both varieties ‘Andes Nec-2’ and ‘Andes Nec-3’ exhibit contrasting susceptibility to mealiness. This will help us to understand the processes underlying the biology of the models we are analyzing, and thus potentially in the future contribute to reduce the occurrence of this physiological disorder that leads to consumer dissatisfaction and food losses. Additionally, by using a metabolomics approach we could elucidate compounds to serve as potential biomarkers that could be used, for example, in breeding programs aimed at obtaining new varieties of peaches and nectarines with longer postharvest lives.

## 2. Results

### 2.1. Phenotypic Properties and Metabolite Analysis of Unripe and Ripe Nectarines at Harvest and after Cold Storage

**Figure 1:**
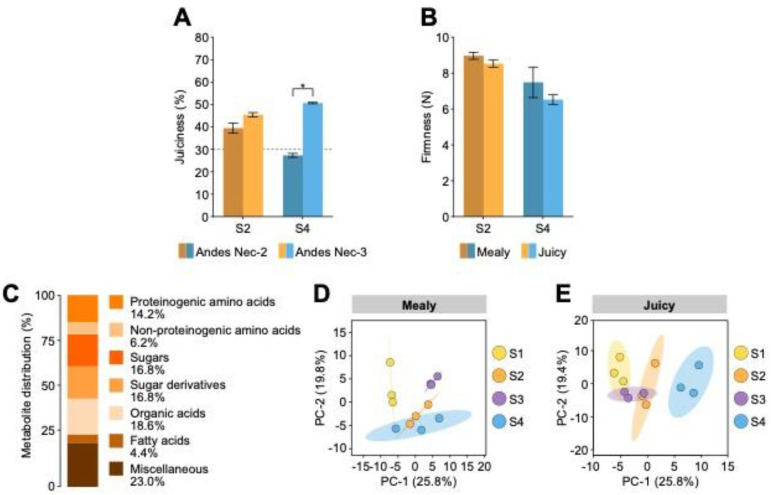
Nectarine phenotyping and global metabolite analysis. (**A**) Juiciness percentage in ripe fruits from Andes Nec-2 and Andes Nec-3 varieties. (**B**) The firmness of ripe fruits from the mealy and juicy varieties. Error bars represent SEM (*n* = 9). Data were tested by *t*-test (* *p* < 0.05). (**C**) Distribution of identified metabolites by GC-MS. Principal Component Analysis (PCA) of metabolites from S1, S2, S3, and S4 stages. Score plots of the mealy metabolite dataset (**D**) and the juicy metabolite dataset (**E**) consisting of three biological replicates (*n* = 3).

Andes Nec-2 and Andes Nec-3 nectarines were studied at different stages (Supplementary Figure S1). First, the physiological parameters of juiciness and firmness were evaluated using ripe nectarines at sampling points S2 and S4. Next, to determine the degree of chilling injury in both varieties, juice percentage was evaluated, which showed no difference in ripe fruit of both varieties at the S2 stage (Figure 1A). Interestingly, after cold storage, fruit ripening at the S4 stage showed a significant difference in percentage juiciness. As described in the Material and Methods, fruits with less than 30% juice content were considered mealy. Therefore, Andes Nec-2 and Andes Nec-3 correspond to “mealy” and “juicy” varieties, respectively. Additionally, nectarine firmness was measured to confirm pulp softening in ripe fruit, showing no differences in both varieties at stages S2 and S4 (Figure 1B). 

To better understand the metabolic changes associated with mealiness induced by cold storage, a non-targeted metabolite profiling analysis was performed using GC-MS and the relative abundances of polar metabolites were quantified. From this analysis, 111 compounds were identified and classified (Supplementary Table S2), which showed different metabolite distributions, being 14.2% proteinogenic amino acids, 6.2% non-proteinogenic amino acids, 16.8% sugars, 16.8% sugar derivatives, 18.6% organic acids, 4.4% fatty acids and 23.0% miscellaneous compounds (Figure 1C). All compounds were found in all four stages in both varieties, and principal component analysis (PCA) was performed. The score plot for the mealy variety explained 45.6% of the variability with two components (Figure 1D). In contrast, for the juicy variety, the score plot explained 45.2% of the variability with two components (Figure 1E).

### 2.2. Effect of Cold Storage on the Metabolite Profiling of Unripe Nectarines

**Figure 2:**
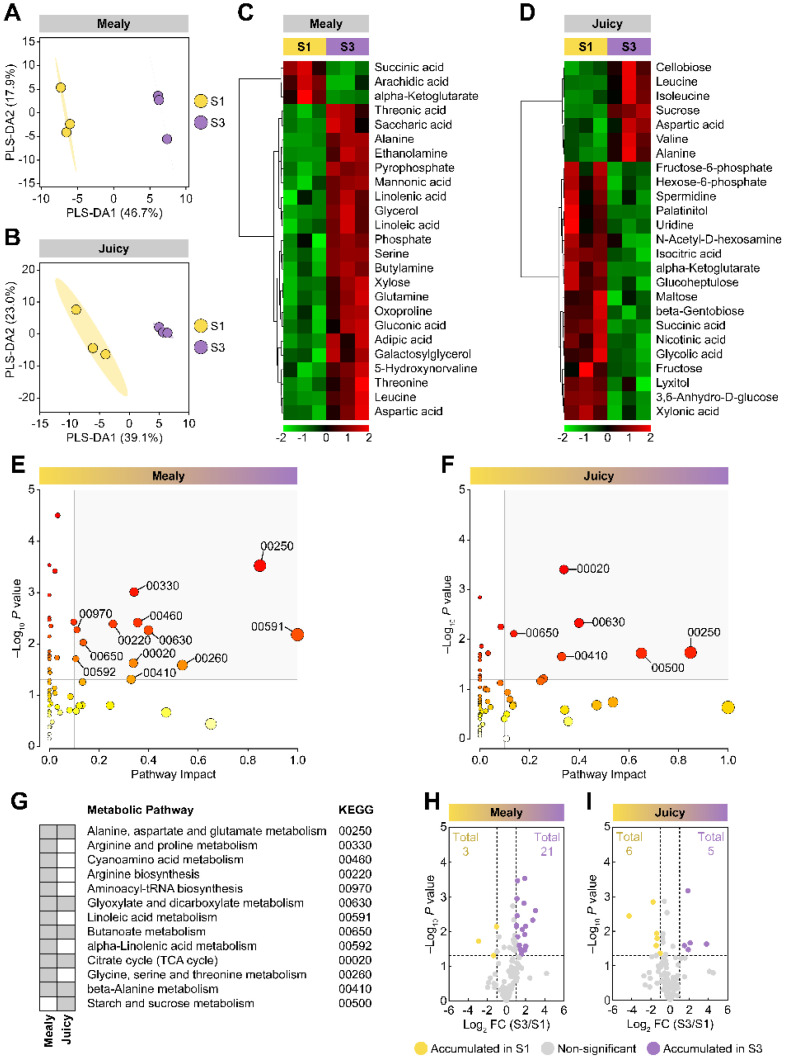
Multivariate analyses of metabolites of mealy and juicy unripe nectarines after cold storage. Partial Least Squares Regression Discriminant Analysis (PLS-DA) for S1 and S3 stages and score plot of mealy (**A**) and juicy (**B**) varieties. The identified metabolites were employed as predictor variables and the varieties as response variables. Heatmap representation based on top 25 metabolites identified by PLS-DA VIP of S1 and S3 stages from the mealy (**C**) and the juicy (**D**) varieties. The columns represent biological replicates for each treatment. The similarity measure used to group the different characteristics was based on Euclidean distance and Ward’s linkage from three biological replicates (*n* = 3). Pathway analysis of S1 and S3 stages from the mealy (**E**) and the juicy (**F**) varieties. Perturbed metabolic pathways and their KEGG entries are highlighted in grey (**G**). Three biological replicates (*n* = 3) and the *Arabidopsis thaliana* pathway library were used for this analysis. Volcano plots showing differentially accumulated metabolites (DAMs) from mealy (**H**) and juicy (**I**) varieties.

A multivariate statistical analysis was carried out using unripe fruits to investigate further the effect of cold storage on metabolic dynamics in nectarines. A PLS-DA analysis using the S1 and S3 stages (of mealy and juicy nectarines) as response variables and the 111 metabolites identified as predictor variables indicated that the stages could be separated. The scores plots accounted for 64.6% (mealy variety) and 62.1% (juicy variety) of the variability with two components (Figure 2A,B). In additional to these findings, data visualization by heatmaps was conducted, displaying the top 25 metabolites based on PLS-DA VIP (Variables Importance in Projection). This profiling showed that compounds are mainly grouped into those with decreased relative amounts (in green) and those with increased relative abundance (in red) in every variety, showing a distinct accumulation of metabolites in the different S1 and S3 stages (Figure 2C,D).

The unripe nectarines from the mealy variety showed that 88% (22/25) of the VIP metabolites were accumulated after cold storage, primarily sugars, sugar derivatives, and amino acids (Figure 2C). On the other hand, the juicy variety displayed a lower number of accumulated VIP metabolites after cold storage (7/25; 28%), primarily amino acids (Figure 2D). Interestingly, cold storage decreased the metabolite abundance of sugars and sugar derivatives in the juicy variety (Figure 2D). A metabolic pathway screening using metabolites linked to a KEGG entry from the mealy (Figure 2E) and juicy variety (Figure 2F) was done. These metabolic pathways were considered perturbed by having many pathway-related metabolites and significant differences in the abundance of these compounds (circles within the gray area) than the other pathways observed in the mealy and juicy varieties. Figure 2G shows the metabolic pathways identified. Curiously, it was found that both varieties shared differential metabolic pathways. Moreover, differentially accumulated metabolites (DAMs) induced by cold storage were analyzed by volcano plots showing that the 21 metabolites strongly accumulated in nectarines from the mealy variety were proteinogenic amino acids, including alanine, serine, leucine, glutamine, proline, isoleucine, and valine. In addition, non-proteinogenic amino acids were elevated, such as 5-hydroxynorvaline and 4-aminobutyric acid (GABA). Other compounds include organic acids, sugars, and sugar derivatives, including mannonic acid, linoleic acid, threonic acid, adipic acid, saccharic acid, cellobiose, fucose, epicatechin, and stigmasterol, which were also accumulated after cold storage. Additionally, α-ketoglutarate, succinic acid, and uridine were diminished following cold storage (Figure 2H). For unripe juicy nectarines, cold storage induced the accumulation of several proteinogenic amino acids, including valine, leucine, alanine, and isoleucine, and the sugar sucrose. Cold storage promoted a decrease in succinic acid and α-ketoglutarate, and other metabolites such as glucoheptulose, β-gentobiose, and palatinitol (Figure 2I).

### 2.3. Effect of Cold Storage on the Metabolite Profiling of Ripe Nectarines

**Figure 3:**
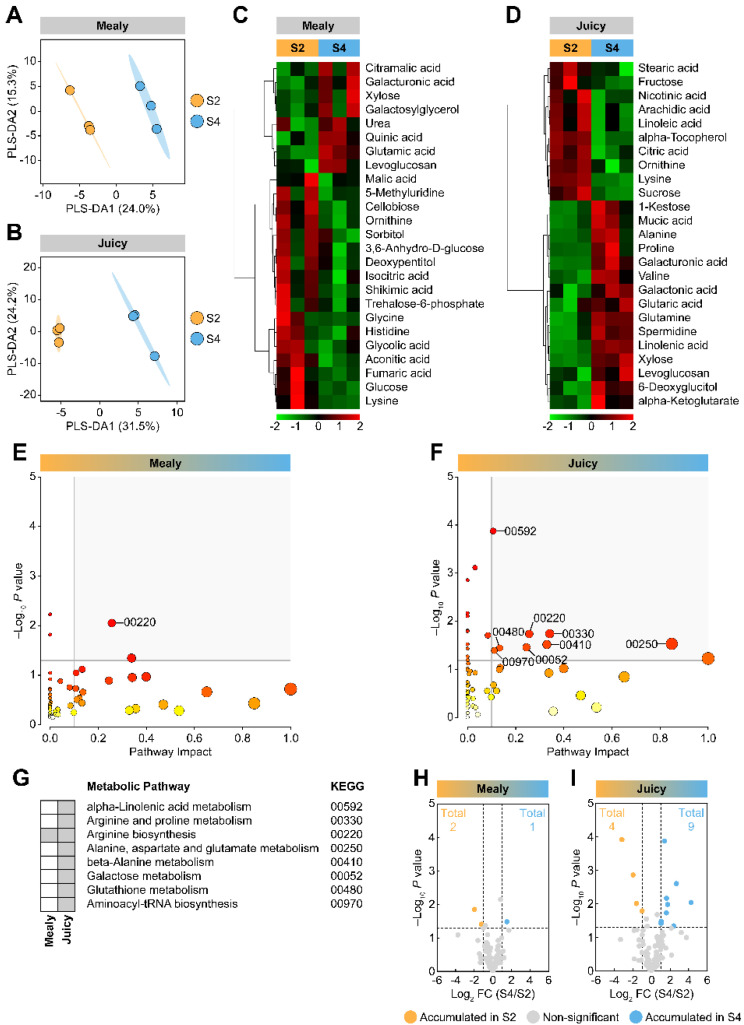
Multivariate analyses of metabolites of mealy and juicy ripe nectarines after cold storage. Partial Least Squares Regression Discriminant Analysis (PLS-DA) for S2 and S4 stages and score plot of mealy (**A**) and juicy (**B**) varieties. The identified metabolites were employed as predictor variables and the varieties as response variables. Heatmap representation based on top 25 metabolites identified by PLS-DA VIP of S2 and S4 stages from the mealy (**C**) and the juicy (**D**) varieties. The columns represent biological replicates for each treatment. The similarity measure used to group the different characteristics was based on Euclidean distance and Ward’s linkage from three biological replicates (*n* = 3). Pathway analysis of S2 and S4 stages from the mealy (**E**) and the juicy (**F**) varieties. Perturbed metabolic pathways and their KEGG entry are highlighted in grey (**G**). Three biological replicates (*n* = 3) and the *Arabidopsis thaliana* pathway library were used for this analysis. Volcano plots showing differentially accumulated metabolites (DAMs) from mealy (**H**) and juicy (**I**) varieties.

For ripe nectarines, the same analyses were conducted. A PLS-DA analysis using the S2 and the S4 stages (from mealy and juicy ripe nectarines) showed that the stages are separated. The score plots explained 39.3% (mealy variety) and 55.7% (juicy variety) of the variability with two components (Figure 3A,B). In addition, visualization of the data using heat maps containing the top 25 metabolites from PLS-DA VIP screening indicated that the compounds showed differential accumulation at S2 and S4 stages. (Figure 3C,D). For the mealy variety, cold storage induced the accumulation of 32% (8/25) of the VIP metabolites, including organic acids, sugars, sugar derivatives, and amino acids (Figure 3C). The juicy variety showed 60% (15/25) of the VIP compounds, mostly amino acids, sugars, and sugar derivatives (Figure 3D). A metabolic pathway analysis for the mealy variety indicated one differentially induced pathway by cold storage (Figure 3E). The metabolic pathway analysis using metabolites from the juicy variety exhibited eight differentially induced pathways by cold storage (Figure 3F). Figure 3G shows the metabolic pathways identified. 

Interestingly, both varieties shared only one differential metabolic pathway, identified as arginine biosynthesis (KEGG 00220). Moreover, differentially accumulated metabolites induced by cold storage were analyzed by volcano plots showing that only one metabolite was strongly accumulated in nectarines from the mealy variety (quinic acid; Figure 3H). Additionally, aconitic acid and cellobiose were diminished following cold storage (Figure 3H). For ripe, juicy nectarines, cold storage induced the accumulation of amino acids, including glutamine, spermidine, and valine, as well as sugars and sugar derivatives such as xylose, galacturonic acid, galactonic acid, and 1-kestose. Cold storage promoted a decrease in citric acid, α-tocopherol, sucrose, and lysine (Figure 3I).

### 2.4. Effect of Cold Storage on the Ripening Process of Nectarines

**Figure 4:**
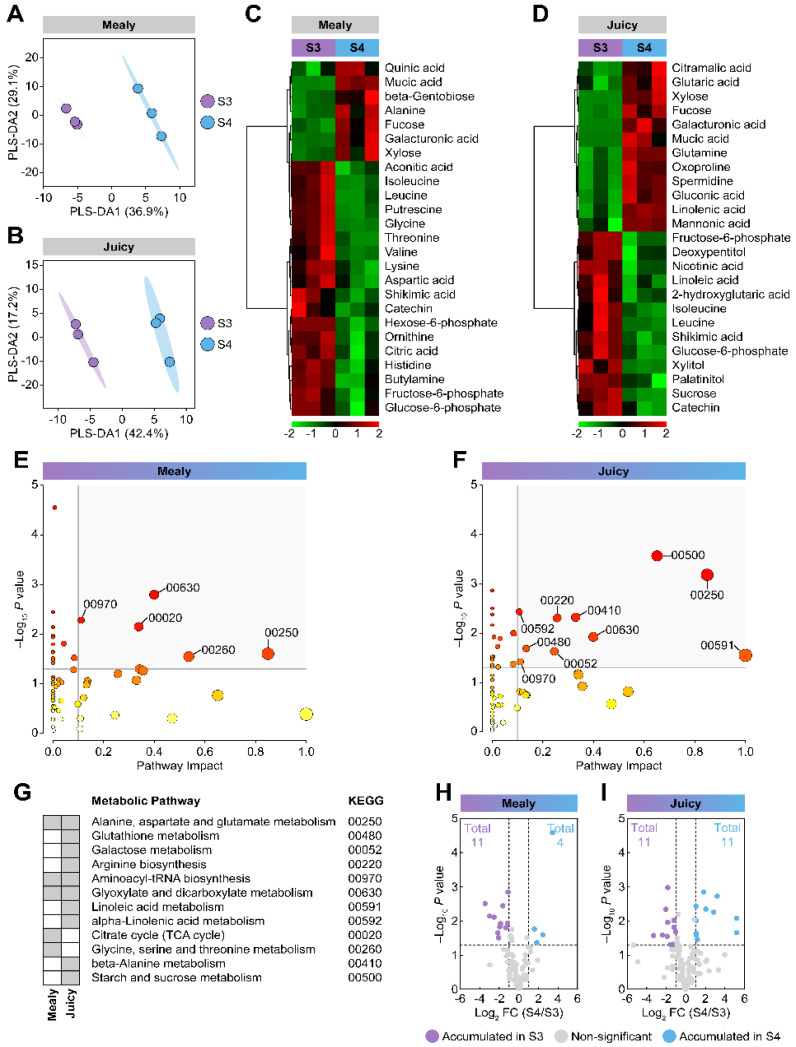
Multivariate analyses of metabolites of mealy and juicy ripening nectarines after cold storage. Partial Least Squares Regression Discriminant Analysis (PLS-DA) for S3 and S4 stages and score plot of mealy (**A**) and juicy (**B**) varieties. The identified metabolites were employed as predictor variables and the varieties as response variables. Heatmap representation based on top 25 metabolites identified by PLS-DA VIP of S3 and S4 stages from the mealy (**C**) and the juicy (**D**) varieties. The columns represent biological replicates for each treatment. The similarity measure used to group the different characteristics was based on Euclidean distance and Ward’s linkage from three biological replicates (*n* = 3). Pathway analysis of S3 and S4 stages from the mealy (**E**) and the juicy (**F**) varieties. Perturbed metabolic pathways and their KEGG entries are highlighted in grey (**G**). Three biological replicates (*n* = 3) and the *Arabidopsis thaliana* pathway library were used for this analysis. Volcano plots showing differentially accumulated metabolites (DAMs) from mealy (**H**) and juicy (**I**) varieties.

Since multiple changes have been found in unripe and ripe nectarines, we investigated the ripening process associated with cold storage. A multivariate analysis was performed on ripening nectarines. A PLS-DA analysis using the S3 and the S4 stages (for mealy and juicy nectarines) as response variables and the metabolites identified as predictor variables showed that stages are separated. The score plots explained 66.0% (mealy variety) and 59.6% (juicy variety) of the variability with two components (Figure 4A,B). In addition, display of the data using heat maps containing the top 25 metabolites based on PLS-DA VIP screening indicated that the compounds exhibited differential accumulation at the S3 and S4 stages (Figure 4C,D). For the mealy variety, cold storage induced the accumulation of 28% (7/25) of the VIP metabolites, including organic acids, sugars, sugar derivatives, and amino acids (Figure 4C). The juicy variety showed an accumulation of 48% (12/25) of the VIP compounds, primarily amino acids, sugars, and sugar derivatives (Figure 4D). A metabolic pathway analysis utilizing metabolites linked to a KEGG entry of the mealy variety pointed to five pathways differentially enhanced by cold storage (Figure 4E). The metabolic pathway analysis using metabolites from the juicy variety displayed ten differentially induced pathways by cold storage (Figure 4F). Additionally, we observed that both varieties differed in their levels of citrate in several metabolic pathways (Figure 4G). The volcano plots of DAMs induced by cold storage displayed the four metabolites strongly accumulated in ripe nectarines from the mealy variety, including sugars and sugar derivatives, such as mucic acid (galactaric acid), galacturonic acid, β-gentobiose, and fucose (Figure 4H). Other metabolites, mainly amino acids such as threonine, histidine, glycine, lysine, valine, leucine, and isoleucine, displayed a decreased accumulation in ripe nectarines from the mealy variety (Figure 4H). Furthermore, in ripe nectarines from the juicy variety after cold storage, eleven increased DAMs were found, including mucic acid, galacturonic acid, gluconic acid, mannonic acid, xylose, fucose, glutaric acid, citramalic acid, and glutamine (Figure 4I). On the other hand, eleven diminished DAMs were observed in juicy ripe nectarines following cold storage, involving palatinitol, xylitol, glucose-6-phosphate, sucrose, fructose-6-phosphate, cyano-L-alanine, isoleucine, and leucine (Figure 4I). 

### 2.5. Comparison of Metabolite Abundance from Primary Metabolism between Mealy and Juicy Nectarines

**Figure 5:**
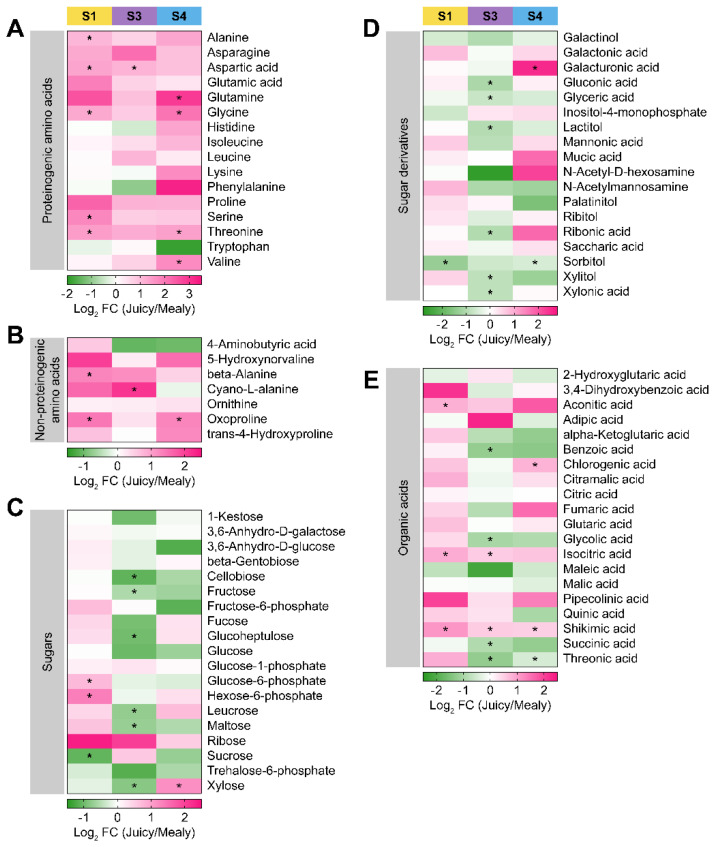
Accumulation of metabolites across nectarine ripening during cold storage. Log_2_ fold change of relative intensity of metabolites from juicy variety over mealy variety in S1, S3, and S4 stages. (**A**) Proteinogenic amino acids, (**B**) non-proteinogenic amino acids, (**C**) sugars, (**D**) sugar derivatives, and (**E**) organic acids. Data were tested by a *t*-test (* *p* < 0.05) comparing mealy and juicy varieties consisting of three biological replicates (*n* = 3).

Because cold storage induced shifts in compounds’ abundance of the primary metabolism of nectarines, we studied the differences between mealy and juicy varieties in accumulating these metabolites across ripening. This analysis found that most proteinogenic amino acids presented a higher accumulation in juicy nectarines than in mealy nectarines (Figure 5A). Alanine, asparagine, aspartic acid, glutamic acid, glutamine, glycine, isoleucine, leucine, proline, serine, threonine, and valine are consistently higher in juicy nectarines across ripening (S1, S3, and S4 stages). In addition, non-proteinogenic amino acids are mainly accumulated in juicy nectarines (Figure 5B); 5-hydroxynorvaline, β-alanine, ornithine, oxoproline, and trans-4-hydroxyproline were higher in juicy than in mealy variety across the ripening process. The accumulation exhibited a heterogeneous pattern for sugars, sugar derivatives, and organic acids (Figure 5C–E). Sugars such as glucose-1-phosphate and ribose showed a consistently higher accumulation during ripening in the juicy variety (Figure 5C). 

On the other hand, 1-kestose, cellobiose, and trehalose-6-phosphate displayed a consistently lower accumulation across ripening in the juicy variety (Figure 5C). For sugar derivatives, only mucic acid was found to be accumulated in juicy nectarines, while galactinol, glyceric acid, and sorbitol were lower in juicy nectarines than in mealy nectarines (Figure 5D). Organic acids, such as aconitic acid, isocitric acid, pipecolinic acid, and shikimic acid, showed increased levels in the juicy than in the mealy variety during ripening (Figure 5E). Otherwise, maleic acid, malic acid, and succinic acid exhibited a consistently lower accumulation across ripening in the juicy variety (Figure 5E).

Moreover, cold storage (S3 and S4 stages) induced interesting shifts in metabolite accumulation. Compounds such as GABA, 3,6-anhydro-D-galactose, 3,6-anhydro-D-glucose, fructose, glucose, glucose-6-phosphate, maltose, lactitol, N-acetylmannosamine, xylitol, α-ketoglutarate, benzoic acid, glycolic acid, and threonic acid presented a major accumulation in the juicy variety at S1 stage, and after cold storage, their abundance increased in the mealy variety (S3 and S4 stages; Figure 5). Furthermore, inositol-4-monophosphate displayed the opposite pattern, showing a decreased accumulation in the juicy variety at the S1 stage, and a lower accumulation in the mealy variety following cold storage (S3 and S4 stages; Figure 5).

### 2.6. Reconstruction of Metabolic Pathways Associated with Primary Metabolism

**Figure 6:**
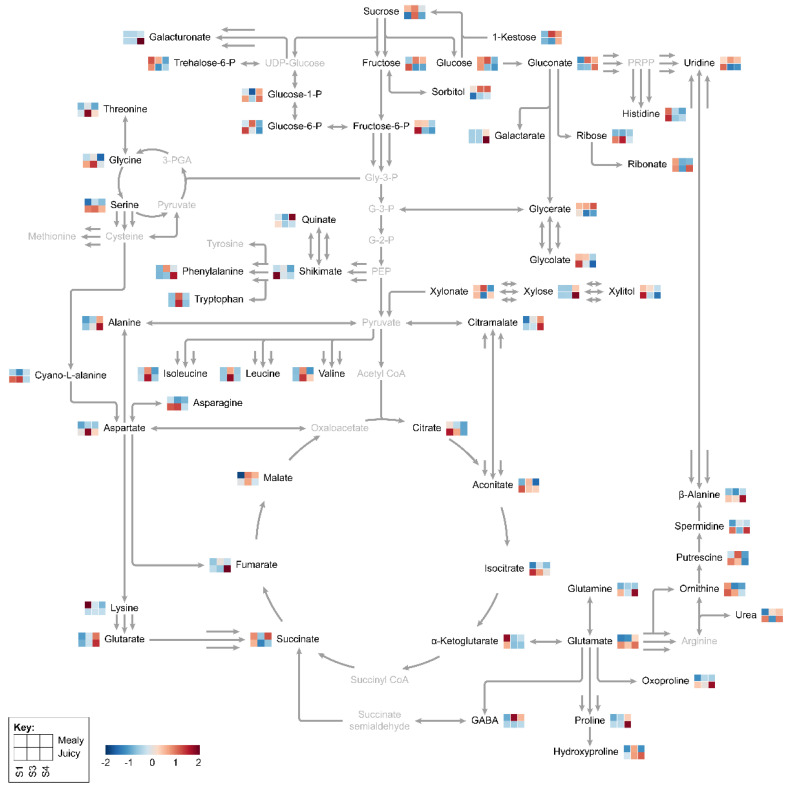
Primary metabolism pathway reconstruction. The map shows the metabolites identified with *p* < 0.05 in black letters, while metabolites not identified or with *p* > 0.05 in grey letters. Relative abundance of metabolites was averaged over three biological replicates (*n* = 3).

The metabolic data analyses indicated that cold storage promoted spatiotemporal changes in primary metabolism (mainly carbohydrate metabolism, TCA cycle, and amino acids metabolisms) throughout unripe and ripe nectarines. Therefore, a metabolic pathway reconstruction was carried out using metabolites with *p* < 0.05 when comparing both mealy and juicy varieties in at least one phenological stage (Figure 6). The map shows metabolites identified with *p* < 0.05 in black letters, while metabolites were not identified in grey letters.

For unripe nectarines from the mealy variety, cold storage induced an upregulation of carbohydrate metabolism. Interestingly, unripe nectarines from the juicy variety exhibited an opposite behavior promoted by cold storage (Figure 6). Related to the TCA cycle, cold storage produced the increase and reduction of different components. Interestingly, only malate was accumulated following cold storage from the juicy variety (Figure 6). In addition, for unripe nectarines from the mealy variety, cold storage induced the accumulation of fifteen amino acids (Figure 6). For juicy unripe nectarines, fourteen amino acids displayed an accumulation following cold storage. A lower abundance was observed for seven amino acids (Figure 6). 

During the ripening of nectarines from the mealy variety, cold storage induced a downregulation of carbohydrate metabolism. Otherwise, ripening nectarines from the juicy and mealy variety exhibited heterogeneous behavior promoted by cold storage (Figure 6). In addition, for ripening nectarines from the mealy variety, cold storage induced the accumulation of seven amino acids. On the other hand, fourteen amino acids were observed in lower levels after cold storage (Figure 6). For juicy ripening nectarines, nine amino acids such as GABA, β-alanine, oxoproline, alanine, glutamic acid, glutamine, histidine, phenylalanine, and proline displayed an accumulation following cold storage (Figure 6). 

## 3. Discussion

In this work, we describe spatiotemporal changes in metabolomic composition across the ripening of nectarines from two varieties with contrasting mealiness induced by cold storage. It is important to note that the concept of mealiness has different definitions depending on the methodology used to quantify it. However, in the current work, a fruit was considered mealy when it presented a juice content lower than 30% [9]. 

It is widely known that carbohydrates are incorporated into the central carbon pathway to produce cellular energy and the biosynthesis of organic acids, amino acids, lipids and secondary metabolites, which are relevant to enable life and development of quality traits highly appreciated by the consumer. In this aspect, we observed rearrangements in primary metabolism associated with cold storage dependent on the variety, suggesting that nectarines ‘Andes Nec-2’ and ‘Andes-Nec-3’ responded with differential signatures of metabolite accumulation. 

### 3.1. Cold Storage Induced Differential Metabolic Rearrangements in Nectarines

Postharvest treatment at 0 °C promoted changes in metabolite accumulation in both varieties. We observed common changes in unripe nectarines from VIP compounds, in which alanine, leucine, and aspartic acid showed a higher accumulation following cold storage. In contrast, succinic acid and α-ketoglutarate were diminished after cold storage independent of the variety. Similar changes were recently described in a segregant nectarine population (‘Venus’ × ‘Venus’) with contrasting mealiness individuals, and α-ketoglutarate, alanine, and leucine shared the same accumulation pattern [26]. Moreover, peach fruits from the juicy varieties ‘Red Globe’ and ‘Limon Marelli’ and the mealy variety ‘Flordaking’ exhibited the same metabolite pattern for succinic acid, α-ketoglutarate, alanine, and aspartic acid [29]. 

Interestingly, unripe nectarines from the mealy variety accumulated more metabolites than the juicy variety after cold storage (19 and 4, respectively). However, when comparing both varieties, threonic acid, saccharic acid, mannonic acid, xylose, gluconic acid, and cellobiose displayed an increased abundance in the mealy variety. At the same time serine, glutamine, oxoproline, 5-hydroxynorvaline, threonine, isoleucine, valine, and sucrose were highly accumulated in the juicy variety. In the case of sucrose, its possible role in ROS suppression has been raised, which could help to reduce some of the problems derived from low temperatures [34]. In addition, authors have pointed out that high levels of sucrose can help maintain membrane integrity and thus lower susceptibility to low temperature [35]. Surprisingly, these data suggested an upregulation of sugars and sugar derivatives in the mealy variety and an upregulation of amino acids in the juicy variety. 

### 3.2. Metabolite Profiling of Juicy and Mealy Ripe Nectarines after Cold Storage

After cold storage (S4 stage), ripe nectarines from the juicy variety exhibited a higher accumulation of fucose, glucoheptulose, glucose-1-phosphate, hexose-6-phosphate, leucrose, ribose, and xylose than nectarines from the mealy variety. Previous work had reported an increased abundance of ribose and xylose in juicy peaches compared to wooly ones from the ‘Spring Lady’ variety [27]. In addition to these observations, we found that fructose, fructose-6-phosphate, glucose, glucose-6-phosphate, and sucrose levels were lower in nectarines from the juicy variety at ripening after cold storage. Thus, concomitant with data previously described for the chilling injury resistant peach varieties ‘Red Globe’ and ‘Limon Marelli’ and the susceptible variety ‘Flordaking’, showing a decreased abundance of fructose and glucose in juicy ripe peaches after cold storage [29]. Interestingly, for the peach variety ‘June Gold’, pre-conditioned fruits presented an increased expressible juice content and a lower abundance of fructose and glucose [33]. For sugar derivatives, we detected increased levels of galactonic acid, galacturonic acid, gluconic acid, inositol-4-monophosphate, mannonic acid, mucic acid, ribitol, ribonic acid, saccharic acid, and xylonic acid in nectarines from the juicy variety. These findings were also observed in juicy fruits from the ‘Spring Lady’ peach, which contained higher abundances of gluconic acid and saccharic acid than in wooly fruits [27]. Concomitant with our data, juicy ‘June Gold’ peaches exhibited an increased accumulation of galacturonic acid than in mealy fruits [33]. In addition, galactinol, glyceric acid, lactitol, sorbitol, and xylitol were found in lower levels in juicy nectarines. In this aspect, juicy ‘Venus’ nectarines showed an accumulation of polygalacturonase [36], which correlate with the increased amount of galacturonic acid in nectarines from the juicy variety, suggesting the hypothetical incorporation of monosaccharides derived from cell wall remodeling associated with flesh softening after cold storage. A previous report using proteomics in ‘O’Henry’ peach fruits susceptible to chilling injury indicated that mealy ripe fruits accumulated isocitrate dehydrogenase and glutamate dehydrogenase following cold storage, which could explain the lower levels of isocitric acid and the accumulation of glutamic acid in the mealy variety of nectarines [17]. 

Interestingly, a decrease in the abundance of sorbitol in juicy peach fruit after cold storage has also been described [27,33]; in relation to this sugar, it has been indicated that it has the capacity to protect against chilling injury, but it is less than that observed in other sugars [35]. Ripe nectarines from the juicy variety also showed increased aconitic acid, chlorogenic acid, citramalic acid, fumaric acid, glutaric acid, isocitric acid, and shikimic acid. Previous works indicated that fumaric acid was increased in juicy peach fruits [29,33]. Otherwise, α-ketoglutarate, benzoic acid, glycolic acid, maleic acid, malic acid, quinic acid, succinic acid, and threonic acid were abundant in nectarines from the mealy variety. A former study also described a benzoic acid and succinic acid increased amount in wooly peach fruits after cold storage [29]. The increase in shikimic acid in juicy fruits makes sense since this compound is very important in the synthesis of phenylpropanoid compounds [37], and several of these metabolites have been described to have the ability to counteract ROS [38], which are produced under conditions of chilling injury. Amino acid accumulation was mainly higher in ripe nectarines from the juicy variety; however, tryptophan, GABA, and cyano-L-alanine were found to increase in nectarines from the mealy variety after cold storage. Our findings are supported by previous works, in which alanine, valine, isoleucine, glycine, serine, threonine, asparagine, glutamic acid, phenylalanine, β-alanine, ornithine, aspartic acid, proline, and trans-4-hydroxyproline displayed an increased abundance in juicy peach fruits. There are studies where it has been proposed that proline would be related to mechanisms that allow tolerance to cold stress associated with decreased protein degradation and osmotic regulation [39]. In this way and coinciding with our results, a higher proline content has been correlated with a lower susceptibility to cold damage in sensitive species such as grapefruit [40], tomato [41], chickpea [42] and bamboo shoots [43]. Likewise, treatments aimed at increasing proline have decreased susceptibility to chilling injury in nectarines [44]. This higher amount of proline in the ripening of the juicy variety is consistent with the higher amount of ornithine in these fruits, since the OAT (ornithine d-aminotransferase) pathway uses ornithine to synthesize proline and is associated with stress conditions, including low temperature, which has been described in plants [39]. 

A proteomic analysis using the ‘Venus’ nectarines [36] indicated that ripe mealy fruits accumulated malate dehydrogenase following cold storage, concomitant with the higher abundance of malic acid detected in nectarines from the mealy variety. The increase of organic acids in the mealy fruits could have been an attempt to access more energy and carbon skeletons through the participation of TCA, to try to repair the deterioration in the structures resulting from the physiological disorder. In many cold-sensitive crops, an increase in respiration rate due to cooling temperatures has been observed [45]. Likewise, the accumulation of GABA described in mealy fruits (S3) could also have been a strategy to increase the amount of energy via the GABA shunt pathway providing TCA intermediates [46] in fruits that were experiencing chilling injury [47]. In this regard, there are other reports where it has been determined that GABA accumulated in the fruits of the woolly peach that could be supporting this assumption [27,29,33]. Since GABA (γ-aminobutyric acid) is considered a relevant player in the regulation of metabolism under stress conditions [48], it can be hypothesized that the timing of GABA accumulation may be an indicator of cold-induced stress in mealy fruits, where GABA was preferentially accumulated after cold storage (S4). In this aspect, researchers have indicated that GABA content in part may regulate the susceptibility to chilling injury in peach trees [49].

Therefore, our results allow us to suggest that both varieties of ‘Andes Nec-2’ and ‘Andes Nec-3’ have differential responses to cold storage through the accumulation of several metabolites that could support tolerance to low temperatures. 

## 4. Materials and Methods

### 4.1. Plant Material and Postharvest Treatments

The experimental trial was conducted on nectarines (*Prunus persica* (L.), var. *nectarina*) varieties ‘Andes Nec-2’ and ‘Andes Nec-3’ during the 2017–2018 growing season at an experimental orchard located in Santiago (33°30′0.86″ S, 70°49′37.4″ W), Metropolitan Region, Chile. Fruits were collected at commercial harvest maturity based on flesh firmness and chlorophyll absorbance index (*I_AD_*) using a portable Vis/NIR Da-Meter (Sinteleia, Bologna, Italy) (S1 stage; unripe nectarines, Supplementary Figure S1). The second pool of harvested fruits was stored at 20 °C for 5–7 d (S2 stage; ripe nectarines). The third pool of fruits was immediately packed and stored at 0 °C (90% relative humidity) for 30 d (S3 stage; unripe nectarines). Then, the fourth pool of fruits was stored at 20 °C for 2–5 d after cold storage (S4 stage; ripe nectarines). In this stage emerges chilling injury symptoms, such as mealiness. Fruits exhibiting 30% or less juice content are considered mealy [9]. 

### 4.2. Phenotypical Analyses

The different quality parameters were evaluated for all stages described above. At each stage, flesh firmness was assessed on both fruit cheeks utilizing a fruit pressure tester with an 8 mm plunger (Effegi, Alfonsine, Italy). Different ranges were considered for each stage: S1: 45–60 N, S2 and S4: 5–18 N, and S3: 40–60 N. At stage S1, fruit weight (g) was determined, soluble solids content (TSS %) was evaluated with a digital refractometer HI 96811 (Hanna Instruments Inc., Woonsocket, MA, USA), and *I_DA_* was measured using a DA-Meter. At stages S2 and S4, juice content (%) was determined using the paper absorption method (PAM) for quantitative juiciness determination [50]. For quality parameters, nine fruits were analyzed per stage (*n* = 9).

### 4.3. GC-MS Metabolite Profiling Analysis of Polar Compounds

Samples from the S1, S2, S3, and S4 stages were used to evaluate the metabolite of both contrasting phenotypes. Total metabolites were extracted as described by Fiehn et al. [51]. Metabolite analysis was performed by gas chromatography–mass spectrometry (GC-MS) and carried out by the NIH West Coast Metabolomics Center. Data were obtained using the protocol described by Fiehn et al. [51]. 

### 4.4. Metabolic Pathway Assessment

Analysis of the metabolic routes of the identified metabolites by GC-MS was conducted by comparing the different stages S1, S2, S3, and S4 of both nectarine varieties using the ‘Pathway Analysis’ tool from the MetaboAnalyst 5.0 software (Xia Lab, McGill University, Quebec, QC, Canada) [52] with categorical classification and using the *Arabidopsis thaliana* pathway library. In addition, metabolites were assayed by its KEGG (Kyoto Encyclopedia of Genes and Genomes) entry [53].

### 4.5. Statistical Analyses

The statistical workflow was conducted as previously reported [54]. Principal component analysis (PCA) and partial least squares regression-discriminant analysis (PLS-DA) were performed on the normalized data set obtained by GC-MS using MetaboAnalyst 5.0 software. Data were mean-centered and weighted by their standard deviations for PCA and PLS-DA tests. PLS-DA analysis was used with metabolites as predictor variables and S1–S4 stages (of both varieties) as response variables. To allocate equal variance, mean-centered variables weighted by standard deviation were used. Important projection variable (VIP) scores were utilized to filter the PLS results to identify relevant traits. The results were examined using Student’s t-test statistical tools (*p* < 0.05) to target metabolites with significant differences among stages of both strains. Pathways that reached cutoff values *p* < 0.05 and pathway impact ≥ 0.1 were considered perturbed [55]. A two-way ANOVA was utilized to compare the means of every metabolite at different stages and strains to perform the reconstruction of metabolic pathways. For phenotypic assays, the Student’s *t*-test was performed using R software version 4.0.2 (Vienna, Austria) with significance set at *p* < 0.05 and was conducted using the ‘agricolae’ package. Experiments were performed using a minimum three biological replicates (*n* ≥ 3). 

## 5. Conclusions

Our findings contributed further evidence about the effects of cold storage in primary metabolism dynamics and nectarine quality at postharvest. Multivariate analyses indicated that cold storage in unripe nectarines induced mainly amino acid accumulation in both varieties. Interestingly, for ripe nectarines, cold storage induced an accumulation of fewer amino acids in both varieties and exhibited an increased abundance of sugars and organic acids. A pathway reconstruction of primary metabolism revealed that in ripe nectarines, cold storage disrupted metabolite abundance in sugar metabolism and the TCA cycle, leading to a differential accumulation of amino acids, organic acids, and sugars in mealy and juicy nectarines that could be driving a variety-dependent cold response.

## Data Availability

Not applicable.

## Supplementary Materials

The following supporting information can be downloaded at: https://www.mdpi.com/article/10.3390/plants12040766/s1, Table S1: Phenotypical properties of nectarines ‘Andes Nec-2’ and ‘Andes Nec-3’ varieties; Table S2: Polar metabolites detected by GC-MS from nectarines ‘Andes Nec-2’ and ‘Andes Nec-3’ varieties. Figure S1: Representative scheme of experimental design and sampling points.
