# Supplementary material for: Metabolite Profiling Reveals the Effect of Cold Storage on Primary Metabolism in Nectarine Varieties with Contrasting Mealiness
Source: Plants (Basel). 2023 Feb 8;12(4):766. doi: 10.3390/plants12040766 (PMC9965640; doi:10.3390/plants12040766)
Supplement: Supplementary file 1 [file plants-12-00766-s001.zip › Supplementary Figure S1.pdf]

Supplementary Figure S1

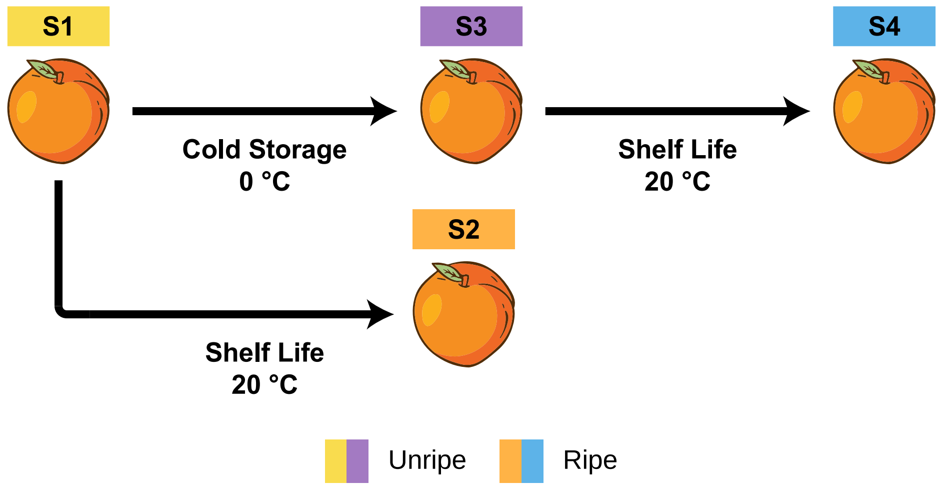

Supplementary Figure S1 : Representative scheme of experimental design and sampling points.
