# Supplementary material for: Metabolite Profiling Reveals the Effect of Cold Storage on Primary Metabolism in Nectarine Varieties with Contrasting Mealiness
Source: Plants (Basel). 2023 Feb 8;12(4):766. doi: 10.3390/plants12040766 (PMC9965640; doi:10.3390/plants12040766)
Supplement: Supplementary file 1 [file plants-12-00766-s001.zip › Supplementary Table S1.pdf]

Supplementary Table S1. Phenotypical properties of nectarines ‘Andes Nec-2’ and ‘Andes Nec-3’ varieties

Stage	Variety	Weight (g)	I_{AD}	TSS %	Firmness (N)	Juice %
S1	Andes Nec-2	269.1	0.77	14.43	62.27	–
	Andes Nec-3	207.6	0.69	13.33	59.68	–
S2	Andes Nec-2	–	–	–	8.97	39.44
	Andes Nec-3	–	–	–	8.53	45.46
S3	Andes Nec-2	–	–	–	59.68	–
	Andes Nec-3	–	–	–	55.04	–
S4	Andes Nec-2	–	–	–	7.47	27.72*
	Andes Nec-3	–	–	–	6.54	50.69

Data were analyzed by t-test (*$P < 0.05$; $n = 9$)
