# Supplementary material for: Metabolite Profiling Reveals the Effect of Cold Storage on Primary Metabolism in Nectarine Varieties with Contrasting Mealiness
Source: Plants (Basel). 2023 Feb 8;12(4):766. doi: 10.3390/plants12040766 (PMC9965640; doi:10.3390/plants12040766)
Supplement: Supplementary file 1 [file plants-12-00766-s001.zip › Supplementary Table S2.pdf]

Supplementary Table S2. Polar metabolites detected by GC-MS from nectarines 'Andes Nec-2' and 'Andes Nec-3' varieties.

Metabolite	Classification	KEGG Entry
1-kestose	Sugar	C03661
1-O-nonyl-lyxitol	Miscellaneous	C01904
2-hydroxyglutaric acid	Organic acid	C02630
3,4-dihydroxybenzoic acid	Organic acid	C00230
3,6-anhydro-D-galactose	Sugar	C06474
3,6-anhydro-D-glucose	Sugar	C06478
4-aminobutyric acid	Non-proteinogenic amino acid	C00334
5-hydroxynorvaline	Non-proteinogenic amino acid	NA
5-methyluridine	Miscellaneous	NA
6-deoxyglucitol	Miscellaneous	NA
aconitic acid	Organic acid	C00417
adipic acid	Organic acid	C06104
alanine	Proteinogenic amino acid	C00041
alpha-ketoglutarate	Organic acid	C00026
alpha-tocopherol	Miscellaneous	C02477
arachidic acid	Fatty acid	C06425
asparagine	Proteinogenic amino acid	C00152
aspartic acid	Proteinogenic amino acid	C00049
benzoic acid	Organic acid	C00539
beta-alanine	Non-proteinogenic amino acid	C00099
beta-gentiobiose	Sugar	C08240
beta-sitosterol	Miscellaneous	C01753
butylamine	Miscellaneous	C18706
catechin	Miscellaneous	C06562
cellobiose	Sugar	C06422
chlorogenic acid	Organic acid	C00852
citramalic acid	Organic acid	C00815
citric acid	Organic acid	C00158
cyano-L-alanine	Non-proteinogenic amino acid	C02512
deoxypentitol	Miscellaneous	NA
diglycerol	Miscellaneous	NA
epicatechin	Miscellaneous	C09727
erythronic acid lactone	Miscellaneous	NA
ethanolamine	Miscellaneous	C00189
fructose	Sugar	C02336
fructose-6-phosphate	Sugar	C00085
fucose	Sugar	C01019
fumaric acid	Organic acid	C00122
galactinol	Sugar derivative	C01235
galactonic acid	Sugar derivative	C00880
galactosylglycerol	Miscellaneous	C05401
galacturonic acid	Sugar derivative	C08348
glucoheptulose	Sugar	C08236
gluconic acid	Sugar derivative	C00257
glucose	Sugar	C00031

glucose-1-phosphate	Sugar	C00446
glucose-6-phosphate	Sugar	C00092
glutamic acid	Proteinogenic amino acid	C00025
glutamine	Proteinogenic amino acid	C00064
glutaric acid	Organic acid	C00489
glyceric acid	Sugar derivative	C00258
glycerol	Miscellaneous	C00116
glycerol-3-phosphate	Miscellaneous	C00093
glycine	Proteinogenic amino acid	C00037
glycolic acid	Organic acid	C03547
hexadecylglycerol	Miscellaneous	C13859
hexose-6-phosphate	Sugar	C02965
histidine	Proteinogenic amino acid	C00135
inositol-4-monophosphate	Sugar derivative	C03546
isocitric acid	Organic acid	C00311
isoleucine	Proteinogenic amino acid	C00407
lactitol	Sugar derivative	D08266
leucine	Proteinogenic amino acid	C00123
leucrose	Sugar	NA
levoglucosan	Miscellaneous	C22350
linoleic acid	Fatty acid	C01595
linolenic acid	Fatty acid	C06427
lysine	Proteinogenic amino acid	C00047
maleic acid	Organic acid	C01384
malic acid	Organic acid	C03668
maltose	Sugar	C00208
mannonic acid	Sugar derivative	C00514
mucic acid	Sugar derivative	C00879
N-acetyl-D-hexosamine	Sugar derivative	C03136
N-acetylmannosamine	Sugar derivative	C00645
nicotianamine	Miscellaneous	C05324
nicotinic acid	Miscellaneous	C00253
ornithine	Non-proteinogenic amino acid	C00077
oxoproline	Non-proteinogenic amino acid	C01879
palatinitol	Sugar derivative	NA
palmitic acid	Fatty acid	C00249
phenylalanine	Proteinogenic amino acid	C00079
phosphate	Miscellaneous	C00009
pipecolinic acid	Organic acid	C00408
proline	Proteinogenic amino acid	C00148
putrescine	Polyamine	C02896
pyrophosphate	Miscellaneous	C00013
quinic acid	Organic acid	C00296
ribitol	Sugar derivative	C00474
ribonic acid	Sugar derivative	C01685
ribose	Sugar	C00121
saccharic acid	Sugar derivative	C00818
serine	Proteinogenic amino acid	C00065

shikimic acid	Organic acid	C00493
sorbitol	Sugar derivative	C00794
spermidine	Polyamine	C00315
stearic acid	Fatty acid	C01530
stigmasterol	Miscellaneous	C05442
succinic acid	Organic acid	C00042
sucrose	Sugar	C00089
threonic acid	Organic acid	C01620
threonine	Proteinogenic amino acid	C00188
trans-4-hydroxyproline	Non-proteinogenic amino acid	C01157
trehalose-6-phosphate	Sugar	C00689
tryptophan	Proteinogenic amino acid	C00078
urea	Miscellaneous	C00086
uridine	Miscellaneous	C00299
valine	Proteinogenic amino acid	C00183
xylitol	Sugar derivative	C00379
xylonic acid	Sugar derivative	C05411
xylose	Sugar	C00181
